# Genetic Progress of Seed Yield and Nitrogen Use Efficiency of Brazilian *carioca* Common Bean Cultivars Using Bayesian Approaches

**DOI:** 10.3389/fpls.2020.01168

**Published:** 2020-08-05

**Authors:** Douglas Mariani Zeffa, Vânia Moda-Cirino, Isabella Arruda Medeiros, Gustavo Henrique Freiria, José dos Santos Neto, Suzana Tiemi Ivamoto-Suzuki, Jéssica Delfini, Carlos Alberto Scapim, Leandro Simões Azeredo Gonçalves

**Affiliations:** ^1^ Área de Genética e Melhoramento Vegetal, Instituto de Desenvolvimento Rural do Paraná, Londrina, Brazil; ^2^ Departamento de Agronomia, Universidade Estadual de Maringá, Maringá, Brazil; ^3^ Laboratório de Ecofisiologia e Biotecnologia Agrícola, Departamento de Agronomia, Universidade Estadual de Londrina, Londrina, Brazil

**Keywords:** *Phaseolus vulgaris* L., plant breeding, abiotic stress, Additive Main effects and Multiplicative Interaction (AMMI), Nitrogen Efficiency Use (NUE)

## Abstract

Common bean (*Phaseolus vulgaris* L.) is one of the most important crops worldwide and is considered an essential source of proteins, fibers, and minerals in the daily diet of several countries. Nitrogen (N) is considered the most important nutrient for common bean crop. On the other hand, the reduction of chemical fertilizers is a global challenge, and the development of cultivars with more N use efficiency (NUsE) is considered one of the main strategies to reduce the amount of N fertilizers. Genetic progress of NUsE has been reported in several crops; however, there was still no quantity in common bean. In this study, our goal was to analyze the genetic progress of seed yield (SY) and NUsE-related traits of 40 *carioca* common bean cultivars release from 1970 to 2017 in eight environments under low (zero) or high N (40 kg ha^−1^) in top-dressing. Genetic progress, principal component analysis, correlations among traits, and cultivar stability were analyzed using Bayesian approaches. The lowest values of the deviance information criterion (DIC) for the full model tested indicated the presence of the genotype × N × environment interaction for all evaluated traits. Nitrogen utilization efficiency (NUtE) and nitrogen uptake efficiency (NUpE) were the traits that most contributed to discriminate cultivars. The genetic progress of SY under high N (0.53% year^−1^, 95% HPD = 0.39; 0.65% year^−1^) was similar to that obtained in low N conditions (0.48% year^−1^, 95% HPD = 0.31; 0.64% year^−1^). These results indicate that modern cultivars do not demand more N fertilizers to be more productive. In addition, we observed a high genetic variability for NUsE-related traits, but there was no genetic progress for these variables. SY showed negative correlation with seed protein content (Prot) in both N conditions, and there was no reduction in Prot in modern cultivars. Both modern and old cultivars showed adaptability and stability under contrasting N conditions. Our study contributed to improve our knowledge about the genetic progress of common bean breeding program in Brazil in the last 47 years, and our data will help researchers to face the challenge of increase NUsE and Prot in the next few years.

## Introduction

Common bean (*Phaseolus vulgaris* L.) is considered the main legume species for human consumption and represents an essential source of proteins, carbohydrates, fibers, and trace minerals in several countries worldwide (Myers and Kmiecik, 2017). Brazil is one of the main world producers and consumers of common beans. In 2019, it produced about 2,360 tons of seeds in an area of 1,619 ha (CONAB, 2020a). Common bean is a basic food used daily in the Brazilian diet with a consumption of 17 kg person^−1^ year^−1^ (Ribeiro et al., 2020). To meet this demand, common beans are grown throughout the all year under different cultivation systems and with different technological levels (Heinemann et al., 2016). In general, Brazilian consumers prefer the Mesoamerican *carioca* (cream seed coat with brown stripes) and black beans, which represent around 70 and 15% of total common bean production, respectively (Pereira et al., 2019).

Although Brazilian common bean production has grown in recent decades, the cultivated area has decreased considerably since 1990 (**Supplementary Figure 1**). This increase in Brazilian production is mainly due to the use of chemical fertilizers, the adoption of new production technologies, and the release of more productive cultivars (Tsutsumi et al., 2015). According to the Ministry of Agriculture, Livestock, and Food Supply (MAPA, 2020), more than 350 bean cultivars are registered in Brazil, developed mainly by public research institutes, such as the Brazilian Agricultural Research Corporation (Embrapa) (Lopes et al., 2012), Instituto de Desenvolvimento Rural do Paraná–IAPAR–EMATER (IDR-Paraná) (Moda-Cirino et al., 2012), and Instituto Agronômico de Campinas (IAC) (Carbonell et al., 2012).

Common bean plants can fix atmospheric nitrogen (N_2_) interacting symbiotically with *Rhizobium* genus bacteria (Appelbaum, 2018). This symbiotic process (biological nitrogen fixation—BNF) is responsible for around 40% and chemical fertilizer for 60% of the total nitrogen (N) demand for this crop (Argaw et al., 2015; Dwivedi et al., 2015). N is the most important macronutrient influencing common bean yield (Barros et al., 2018). Therefore, the development of cultivars that have greater N use efficiency (NUsE) is considered one of the main strategies to reduce the amount of chemical fertilizers in sustainable farming systems (Mueller et al., 2019).

NUsE can be divided into two components (Moll et al., 1982): (i) N utilization efficiency (NUtE) which is the capacity of the plant to convert N into seed biomass; and (ii) N uptake efficiency (NUpE) which means the capacity of the plant to absorb N from the soil. In legume crops, NUpE is based on the N acquisition from the soil and efficiency of the BNF process (Diacono et al., 2019). Several studies have already described the importance of BNF for common beans; however, studies related to NUsE components are still scarce (Farid et al., 2017; Kamfwa et al., 2019).

Information on the quantification of genetic progress for any crop is important for plant breeders, allowing them to design future breeding program strategies (Stahl et al., 2017; de Faria et al., 2018). In Brazil, genetic progress studies related to common bean seed yield have been extensively reported (Chiorato et al., 2010; de Faria et al., 2013; Barili et al., 2016), but there is no study combining information on the genetic progress of seed yield and NUsE under N contrasting conditions. In addition, a negative correlation between seed yield and protein content (Prot) may have caused its decrease over the years as reported in wheat (Laidig et al., 2017). In the face of this challenge, the question arises whether or not breeding is directing NUsE and Prot improvement.

Bayesian methods have been increasingly used in plant breeding studies (da Silva et al., 2020; Nascimento et al., 2020). The fundamental idea of the Bayesian approach is to describe all uncertainty and variation using probability distributions. Bayesian analysis offers the ability to utilize data containing unbalanced structures, select and study complex models, obtain more precise credibility intervals, and incorporate *a priori* information (Aczel et al., 2020). In this way, our main goal was to quantify the genetic progress of seed yield and NUsE-related traits in Brazilian *carioca* common bean cultivars using Bayesian approaches. Our hypothesis was that modern cultivars are more responsive and have improved NUsE when compared to cultivars released in the past since breeding programs are evaluating and selecting superior genotypes under N top-dressing.

## Material and Methods

### Plant Material

Forty Brazilian *carioca* common bean cultivars released from 1970 to 2017 and registered in the National Register of Cultivars of the Brazilian Ministry of Agriculture, Livestock, and Food Supply (RNC-MAPA) were evaluated (**Table 1**). These cultivars represent a large part of the genetic variability that exists between *carioca* common bean cultivars grown during the approximately 50 years of history of *carioca* common bean breeding in Brazil. Seed samples were obtained from the Gene Bank of the Instituto de Desenvolvimento Rural do Paraná (IDR-Paraná), Londrina, Brazil, and subsequently multiplied in order to standardize seed germination. Information about the genealogy of the cultivars is available in **Supplementary Table 1**.

**Table 1:** Characteristics of all 40 *carioca* common bean cultivars analyzed in this study.

Code	Cultivar	Release year	Origin^1^	Grown habit	Architecture
1	Carioca	1970	IAC	Indeterminate	Prostrate
2	IAPAR 14	1986	IDR-Paraná	Indeterminate	Semierect
3	IAPAR 57	1989	IDR-Paraná	Indeterminate	Semierect
4	IAPAR 72	1994	IDR-Paraná	Indeterminate	Prostrate
5	Pérola	1996	Embrapa	Indeterminate	Semierect
6	IAPAR 80	1997	IDR-Paraná	Indeterminate	Semierect
7	IAPAR 81	1997	IDR-Paraná	Indeterminate	Erect
8	FTS Bonito	1998	FT Sementes	Indeterminate	Semierect
9	ANFc 9	1998	Agro Norte	Indeterminate	Semierct
10	Princesa	1998	Embrapa	Indeterminate	Semierect
11	IPR Juriti	2002	IDR-Paraná	Indeterminate	Erect
12	BRS Talismã	2002	Embrapa	Indeterminate	Semierect
13	BRS Pontal	2003	Embrapa	Indeterminate	Erect
14	BRS Requinte	2003	Embrapa	Indeterminate	Semierect
15	IPR Saracura	2004	IDR-Paraná	Indeterminate	Semierect
16	IPR Colibri	2004	IDR-Paraná	Determinate	Erect
17	BRS Horizonte	2004	Embrapa	Indeterminate	Erect
18	BRSMG Pioneiro	2005	Embrapa	Indeterminate	Erect
19	IPR Eldorado	2006	IDR-Paraná	Indeterminate	Semierect
20	IPR 139	2007	IDR-Paraná	Indeterminate	Semierect
21	IAC Alvorada	2007	IAC	Indeterminate	Semierect
22	IPR Tangará	2008	IDR-Paraná	Indeterminate	Erect
23	FTS 65	2008	FT Sementes	Indeterminate	Semierect
24	BRS Estilo	2009	Embrapa	Indeterminate	Erect
25	TAA Bola Cheia	2009	Terra Alta	Indeterminate	Prostrate
26	IPR Campos Gerais	2011	IDR-Paraná	Indeterminate	Erect
27	IPR Andorinha	2012	IDR-Paraná	Determinate	Semierect
28	BRSMG Madrepérola	2012	Embrapa	Indeterminate	Prostrate
29	BRS Ametista	2012	Embrapa	Indeterminate	Semierect
30	IPR Curió	2013	IDR-Paraná	Determinate	Erect
31	BRS Notável	2013	Embrapa	Indeterminate	Erect
32	IAC Imperador	2013	IAC	Determinate	Erect
33	TAA Gol	2013	Terra Alta	Determinate	Erect
34	IPR Maracanã	2013	IDR-Paraná	Indeterminate	Erect
35	TAA Dama	2013	Terra Alta	Indeterminate	Prostrate
36	IPR Quero-quero	2014	IDR-Paraná	Indeterminate	Erect
37	IPR Bem-te-vi	2014	IDR-Paraná	Indeterminate	Erect
38	IPR Celeiro	2016	IDR-Paraná	Indeterminate	Erect
39	IAC Sintonia	2016	IAC	Indeterminate	Semierect
40	IPR Sabiá	2017	IDR-Paraná	Indeterminate	Erect

^1^IDR-Paraná: Instituto de Desenvolvimento Rural do Paraná; Embrapa, Brazilian Agricultural Research Corporation; IAC, Instituto Agronômico de Campinas.

### Experimental Design

Cultivars were evaluated at the Experimental Stations of IDR-Paraná in Londrina and Ponta Grossa during the rainy season of 2017 and in Santa Tereza do Oeste and Ponta Grossa in the dry season of 2018. Soil physical–chemical analyses and other characteristics related to the assessment sites are shown in **Table 2**. The experiments were arranged in a randomized complete block design with four replications. Sowing was carried out mechanically, and the experimental plots consisted of four 4-m rows, spaced 0.5 m apart, with 15 seeds per linear meter. Two levels of N top-dressing were used in each environment, totaling eight independent experiments. Experiments under high N were fertilized with 40 kg N ha^−1^ (urea form) at V_3_ stage development, while the experiments under low N did not receive N top-dressing. In all experiments, the crops were fertilized at sowing with 300 kg ha^−1^ of NPK (04–30–10).

**Table 2:** Location, codes, climate, and soil characterization of four environments evaluated in this study.

Characteristics^1^	Londrina(rainy season of 2017)	Ponta Grossa(rainy season of 2017)	Ponta Grossa(dry season of 2018)	Santa Tereza do Oeste(dry season of 2018)
Code	LD17	PG17	PG18	STO18
Geographical coordinates	23°17′34″S; 51°10′24″W	25°5′40″S; 50°9′48″W	25°5′40″S; 50°9′48″W	25°3′10″S; 53°37′39″W
Altitude (m)	550	956	956	749
Climate^2^	Cfa	Cfb	Cfb	Cfa
Soil	Dystroferric Red Latosol	Dystrophic Red Latosol	Dystrophic Red Latosol	Dystroferric Red Latosol
Sand (%)	79.68	71.56	67.40	71.81
Silt (%)	10.70	14.20	16.74	11.02
Clay (%)	9.62	14.24	15.86	17.17
pH (H_2_0)	5.35	4.77	4.33	5.43
H + Al (cmol_c_ dm^3^)	4.16	7.51	9.56	5.31
K (cmol_c_ dm^3^)	0.34	0.28	0.29	0.39
Ca (cmol_c_ dm^3^)	3.49	4.82	3.31	7.44
Mg (cmol_c_ dm^3^)	2.41	1.55	1.68	2.85
Al (cmol_c_ dm^3^)	0.11	0.20	0.58	0.02
P (mg dm^3^)	14.93	5.92	8.51	9.66
Organic matter (%)	2.18	2.93	2.87	2.21

^1^Physical–chemical analyses were performed using soil layer samples from 0 to 20 cm.

^2^Köppen climate classification = Cfa, Humid subtropical climate; Cfb, Temperate oceanic climate.

### Agronomic Trait Analysis

Five uniform and representative plants were collected at physiological maturation (R_9_ development stage) from each experimental plot. Seeds and aerial part (stems, leaves, and pod shells) were dried in an oven at 70°C for 72 h to determine later the seeds and shoot dry biomass. These samples were also crushed separately using a Willey MA340 type knife mill (Marconi Laboratory Equipment, Piracicaba, Brazil) to determine seed and shoot N content by the Kjeldahl method (Horwitz, 1975) using a Tecnal TE-0371 digester (Tecnal Scientific Equipment, Piracicaba, Brazil). Seed protein content (Prot, %) was determined by multiplying the percentage of N in the seeds by the factor 6.25 (Horwitz, 1975), while harvest index (HI) was defined by the ratio of shoot dry biomass and total dry biomass (seeds and shoot dry biomass).

Seed yield (SY, kg ha^−1^ with moisture of 13%) was obtained after manual removal and mechanical threshing of plants from the two central rows of each plot. NUsE components were determined according to Moll et al. (1982). NUpE (mg per g of N absorbed in each plot) was calculated by the ratio between plant total N content and total N fertilizer applied at sowing and top-dressing. NUtE (g of seeds produced per mg N absorbed) was calculated using the ratio between seed dry biomass and plant total N content, while NUsE (in g of seeds produced per g of N applied in each plot) was determined by the product between NUpE and NUtE. The traits Prot, HI, NUsE, NUtE, and NUpE were evaluated only in Londrina and Ponta Grossa during the rainy season of 2017 under high and low N conditions, while the SY was determined in all experiments.

### Fitting Models

The traits were evaluated by comparing the following models: (i) Full model: considering triple interaction among genotype (G) × nitrogen (N) × environment (E); (ii) Reduced model 1: considering double interaction between G × N; (iii) Reduced model 2: considering double interaction between G × E; and (iv) Null model: considering interaction absence among factors G, N, and E. The Full model follows the mathematical model below:

$$y_{ijkm}=\mu +g_{i}+b_{j/k/m}+e_{k}+n_{m}+ge_{ik}+gn_{im}+en_{km}+gen_{ikm}+\epsilon_{ijkm}$$

Where: *µ* is the overall mean, *g_i_* is the random effect of genotype *i*, *b_j/k/m_* is the random effect of block *j* within environment *k* and within N fertilization *m*, *e_k_* is the fixed effect of environment *k, n_m_* is the fixed effect of N fertilization *m*, *ge_ik_* is the random effect of interaction between genotype × environment, *gn_im_* is the random effect of interaction between genotype × N fertilization, *en_km_* is the fixed effect of interaction between environment × N fertilization, *gen_ikm_* is the random effect of interaction among genotype × environment × N fertilization, and *ϵ_ijkn_* ~ N(0, *σ*²) is the random effect of error associated with each experimental plot.

The marginal *a posteriori* distributions were performed considering non-informative *a priori* distributions for all model parameters using R software (https://www.r-project.org/) through the ‘MCMCglmm’ (Hadfield, 2010) package. A total of 1,000,000 values were generated by MCMC (Monte Carlo Markov Chain) process, assuming a burn-in period and thinning interval of 500,000 and five iterations, respectively. The MCMC convergence was verified using the Heidelberger and Welch (1983) criterion through the ‘coda’ package (Plummer et al., 2006).

The models tested were compared by deviance information criterion (DIC) as proposed by Spiegelhalter et al. (2002): $DIC=D(\bar{\theta })+2p_{D},$where $D(\bar{\theta })$ is a point estimate of the deviance obtained by replacing the parameters with their *a posterior* means estimates in the likelihood function, and *p_D_* is the effective number of parameters in the model. Models with smaller DIC should be preferred to models with higher DIC. However, differences (*D*) between DIC values of models *a* and *b* are given by *D* = | *DIC_a_* – *DIC_b_* |, and thus, if *D* < 5, there is no significant difference among the compared models; if 5 ≤ *D* ≤ 10, the difference is significant; and if *D* > 10, the difference is highly significant.

### Correlations and Principal Component Analysis

Correlations and principal component analysis (PCA) were performed using ‘BayesianFirstAid’ (Bååth, 2014) and ‘bPCA’ (Smyčka and Keil, 2020) packages, respectively. In both analyses, the median scores were reported with their respective 95% highest posterior density (HPD) intervals. Correlation estimates were considered significant when the HPD intervals did not overlap zero. The marginal *a posteriori* distributions were performed considering non-informative *a priori* distributions for all model parameters. A total of 100,000 values were generated by MCMC (Monte Carlo Markov Chain) process, assuming a burn-in period and thinning interval of 10,000 and 10 iterations, respectively. The MCMC convergence was verified using the Heidelberger and Welch (1983) criterion through the ‘coda’ package (Plummer et al., 2006).

### Genetic Progress

Genetic progress was calculated for each trait evaluated in both N conditions using the following simple linear regression model:

$$y_{i}=\beta_{0}+\beta_{1}X_{i}+\epsilon_{i}$$

Where: *β*_0_ is the intercept or linear coefficient, *β*_1_ is the slope or angular coefficient to adjust the data of variable *y*_i_ as a function of *X*_i_ (year of cultivar release), and *ϵ_i_* are errors normally distributed with mean zero and variance *σ*², that is *ϵ_i_* ~ N(0, *σ*²). The parameter *σ*² represents a variability in which the results differ from their predictions based on the model. This model can also be described as *y*_i_ ~ Normal (*β*_0_ + *β*_1_ *X*_i_ + *σ*²). The difference between genetic progresses (Δ*β*_1_) at high and low N fertilization was considered significant when there was no overlap between their HPD intervals.

The marginal *a posteriori* distributions were performed considering non-informative *a priori* distributions for all model parameters using the ‘MCMCglmm’ (Hadfield, 2010) package. A total of 100,000 values were generated by MCMC (Monte Carlo Markov Chain) process, assuming a burn-in period and thinning interval of 100,000 and 10 iterations, respectively. The MCMC convergence was verified using the Heidelberger and Welch (1983) criterion through the ‘coda’ package (Plummer et al., 2006).

### Adaptability and Stability Analyses

Bayesian additive main effects and multiplicative interaction (BAMMI) method was used to study cultivar stability following the model below:

$$y=1_{n}\mu +X_{1}\tau +X_{2}\delta +\sum_{k=1}^{t}\lambda_{k}diag(X_{1}\alpha_{k})X_{2}\gamma_{k}+\epsilon$$

Where: 1*_n_* is the vector of the order *n* × 1, *µ* is the overall mean, *X*_1_ is the matrix of genotypes of order *n* × *g*, *τ* is the effect vector *g* × 1 for genotypes, *X*_2_ and *δ* are the matrices for environments of the order *n* × *a* and the effect vector *a* × 1 for environment, respectively. *λ_k_* is the singular value for the k^th^ principal component, *t* is the number of multiplicative terms [*t* ≤ min (*g*, *a*) − 1), *α_k_* and *γ_k_* are the singular vectors of *k* for genotypes and environment, respectively; and *ϵ* is the vector *n* of error effect. Vector *ϵ* has a multivariate normal distribution with zero mean and variance–covariance matrix $\sigma_{\epsilon }^{2}I_{n}$ Thus, the vector *y* also has a multivariate normal distribution.

For the BAMMI model, the estimation of the parameters of the above equation model assumes that the conditional distribution of *y*, given that *µ*, *τ*, *δ*, *λ*, *α*, *γ*, and $\sigma_{\epsilon }^{2}$ is a multivariate normal distribution:

$$y|\mu ,\tau ,\delta ,\lambda ,\alpha ,\gamma ,\sigma_{\epsilon }^{2}∼N\left(1_{n}\mu +X_{1}\tau +X_{2}\delta +\sum_{k=1}^{t}\lambda_{k}\;diag\;(X_{1}\alpha_{k})X_{2}\gamma_{k}I_{n}\sigma_{\epsilon }^{2}\right)$$

Where: *I_n_* is the identity matrix of order *n*. The *a priori* distributions used for the parameters were the same as those presented by Crossa et al. (2011). Subscripted symbols *μ* and *σ* ^2^ denote mean and variance of the *a priori* distribution of whatever parameter is shown as subscript:

$$\mu |\mu_{\mu },\sigma_{\mu }^{2}∼N(\mu_{\mu },\sigma_{\mu }^{2});$$

$$\tau |\mu_{\tau },\sigma_{\tau }^{2}∼N(\mu_{\tau },I_{g}\sigma_{\tau }^{2});$$

$$\delta |\mu_{\delta },\sigma_{\delta }^{2}∼N(\mu_{\delta },I_{g}\sigma_{\delta }^{2});$$

$\lambda_{k}|\mu_{\lambda_{k}},\sigma_{\lambda_{k}}^{2}∼N^{+}(\mu_{\lambda_{k}},I_{e}\sigma_{\lambda_{k}}^{2})$ with the restrictions *λ_k_* > 0 and *λ_k–_*_1_ ≥ *λ_k_*;

σ*_k_* ~ spherical uniform distribution on the corrected subspace;
γ*_k_* ~ spherical uniform distribution on the corrected subspace; and

$$\sigma_{e}^{2}|v_{e^{\prime \prime }}s_{e}^{2}∼Inv-Scaled-\chi^{2}(v_{e},S_{e}^{2})$$

Where: *N* denotes the normal distribution, *N^+^* is the positive normal distribution, and Inv − Scaled − *χ* ^2^ is the inverse χ^2^ distribution. In this study, the *a priori* distributions were non-informative. The value zero was used as prior distribution for the mean in all genotypic and environmental effects and high values for the variances, resulting in: *µ_µ_* = 0, *µ_τ_* = 1*_g_* × *0*, *µ_δ_* = 1*a* × *0* and *µ_λk_* = 0, and for the variances $\sigma_{\mu }^{2}$,$\sigma_{\tau }^{2}$,$\sigma_{\delta }^{2}$ e $\sigma_{\lambda_{k}}^{2}$ = 1 × 10^15^.

The *a posteriori* distribution was estimated by:

$$p\;(\mu ,\tau ,\lambda ,\alpha ,\gamma ,\sigma_{\epsilon }^{2}|y)$$

$$\propto \;\exp\left[-\;(1/2\;\sigma_{\mu }^{2})(\mu_{\mu }-\mu {)}^{\prime }(\mu_{\mu }-\mu )\right]$$

$$\text{x}\;\text{exp}\left[-\;(1/2\;\sigma_{\tau }^{2})(\mu_{\tau }-\tau {)}^{\prime }(\mu_{\tau }-\tau )\right]$$

$$\text{x}\;\text{exp}\left[-\;(1/2\;\sigma_{\delta }^{2})(\mu_{\delta }-\delta {)}^{\prime }(\mu_{\delta }-\delta )\right]$$

$$\text{x}\;\prod_{k=1}^{t}\text{exp}\;\left[-\;(1/2\sigma_{\lambda }^{2})\;(\mu_{\lambda }-\lambda_{k}{)}^{\prime }(\mu_{\lambda }-\lambda_{k})\right]$$

$$\begin{array}{c} \text{x}\;\text{exp}\;\{-(1/2\sigma_{\epsilon }^{2})[y-1_{n}\mu +X_{1}\tau +X_{2}\delta +\sum_{k=1}^{t}\lambda_{k}\;diag\;(X_{1}\alpha_{k})X_{2}\gamma_{k})\prime [y-1_{n}\mu \\ +X_{1}\tau +X_{2}\delta +\sum_{k=1}^{t}\lambda_{k}\;diag(X_{1}\alpha_{k})X_{2}\gamma_{k}) \end{array}$$

$$\text{x}\left\{{\left(\sigma_{\epsilon }^{2}\right)}^{-\left\{\left[\frac{\left(n+\upsilon_{\epsilon }\right)}{2}\right]-1\right\}}\text{exp[}-(1/2\sigma_{\epsilon }^{2})\upsilon_{\epsilon }s_{\epsilon }^{2}]\right\}$$

A total of 1,000,000 values were generated by MCMC (Monte Carlo Markov Chain) process, assuming a burn-in period and thinning interval of 100,000 and five iterations, respectively. The MCMC convergence was verified using the Heidelberger and Welch (1983) criterion through the ‘coda’ package (Plummer et al., 2006). The BAMMI method was carried out using R script developed by Crossa et al. (2011).

## Results

### Fitted Models and Influence of Nitrogen Fertilization

DIC results showed a positive evidence of a triple interaction among genotype, environment, and N levels (G × E × N) for all evaluated traits since the full model presented lower DIC values (**Table 3**). However, for trait HI, the DIC values difference among full, reduced 1, and reduced 2 models was less than five, indicating that there is no difference among all models tested.

**Table 3:** Deviance information criterion (DIC) for full model [considering interactions among genotype (G) × nitrogen (N) × environment (E)], reduced model 1 (G × N), reduced model 2 (G × E), and null model (considering only additive effects among G, N, and E).

Traits^1^	DIC			
	Full	Reduced 1	Reduced 2	Null
SY (kg ha^−1^)	9,635.3	9,646.7	9,645.6	1,0045.8
NUsE (g g^−1^)	123.55	131.3	139.9	654.5
NUpE (mg g^−1^)	1,198.3	1,296.1	1,297.3	1,720.2
NUtE (g mg^−1^)	867.8	882.0	883.2	913.4
Prot (%)	2,720.7	2,735.7	2,736.5	2,938.3
HI	1,374.6	1,375.0	1,375.8	1,452.5

^1^SY, seed yield; NUsE nitrogen use efficiency; NUpE, nitrogen uptake efficiency; NUtE, nitrogen utilization efficiency; Prot, seed protein content; and HI, harvest index.

The overall mean for all evaluated traits and their respective HPD intervals are shown in **Table 4**. In general, there was a reduction of SY under low N conditions. However, there was an increase in NUsE and NUpE under low N when compared to high N conditions. There were no differences evident between high and low N conditions for the NUtE, Prot, and HI traits since there was an overlap of the HPD intervals in both N conditions. The performance of cultivars is presented in **Supplementary Tables 2–4**.

**Table 4:** Overall means and their respective 95% highest posterior density (HPD) intervals of six traits under low and high N conditions.

Traits^1^	High N		Low N	
	Mean	95% HPD	Mean	95% HPD
SY (kg ha^−1^)	2,495.61	(2,388.95; 2,609.10)	2,123.68	(1,995.85; 2,237.66)
NUsE (g g^−1^)	0.32	(0.21; 0.48)	0.77	(0.56; 1.04)
NUpE (mg g^−1^)	0.12	(0.08; 0.16)	0.28	(0.23; 0.33)
NUtE (g mg^−1^)	2.51	(2.26; 2.75)	2.68	(2.34; 2.86)
Prot (%)	20.21	(18.73; 20.98)	18.87	(17.71; 20.00)
HI	0.46	(0.39; 0.53)	0.45	(0.38; 0.53)

^1^SY, seed yield; NUsE, nitrogen use efficiency; NUpE, nitrogen uptake efficiency; NUtE, nitrogen utilization efficiency; Prot, seed protein content; and HI, harvest index.

### Correlation and Principal Component Analysis

Correlation coefficients (*r*) and their respective HPD intervals among traits evaluated are shown in **Table 5**. In general, there was a moderate agreement between the correlations of high and low N conditions. SY, HI, and NUtE showed a positive correlation in both N levels. On the other hand, SY, HI, and NUtE showed a negative correlation with Prot, which correlated positively with NUsE (*r* = 0.47, 95% HPD = 0.36; 0.58), and NUpE (*r* = 0.54, 95% HPD = 0.46; 0.62) under low N. NUsE showed a positive correlation with NUpE under low (*r* = 0.98, 95% HPD = 0.97; 0.99) and high N (*r* = 0.87, 95% HPD = 0.84; 0.90) conditions. In addition, we observed a positive and negative correlations between NUsE and NUtE under high (*r* = 0.23, 95% HPD = 0.12; 0.34) and low N (*r* = −0.33, 95% HPD = −0.44; −0.22) levels, respectively.

**Table 5:** Correlation coefficients and their respective 95% highest posterior density (HPD) intervals among the traits seed yield (SY), nitrogen use efficiency (NUsE), nitrogen uptake efficiency (NUpE), nitrogen utilization efficiency (NUtE), seed protein content (Prot), and harvest index (HI) under high N (top diagonal) and low N (bottom diagonal).

Traits^1^	SY	NUsE	NUpE	NUtE	Prot	HI
SY		0.04 (−0.08; 0.15)	−0.03 (−0.14; 0.08)	0.27 (0.17; 0.28)	−0.13 (−0.25; −0.02)	0.32 (0.21; 0.43)
NUsE	0.12 (0.02; 0.26)		0.87 (0.84; 0.90)	0.23 (0.12; 0.34)	−0.08 (−0.19; 0.04)	−0.13 (−0.25; −0.18)
NUpE	−0.03 (−0.13; 0.11)	0.98 (0.97; 0.99)		−0.10 (−0.22; 0.01)	0.13 (−0.01; 0.25)	−0.34 (−0.45 −0.22)
NUtE	0.48 (0.39; 0.57)	−0.33 (−0.44; −0.22)	−0.47 (−0.56; −0.38)		−0.70 (−0.76; −0.63)	0.59 (0.50; 0.66)
Prot	−0.38 (−0.48; −0.29)	0.47 (0.36; 0.58)	0.54 (0.46; 0.62)	−0.67 (−0.70; −0.60)		−0.35 (−0.46; −0.25)
HI	0.48 (0.39; 0.56)	−0.63 (−0.70; −0.55)	−0.69 (−0.75; −0.62)	0.70 (0.63; 0.76)	−0.40 (−0.30; −0.50)	

^1^Correlation coefficients are considered as significant when HPD intervals do not overlap the value of zero.

**Figure 1:**
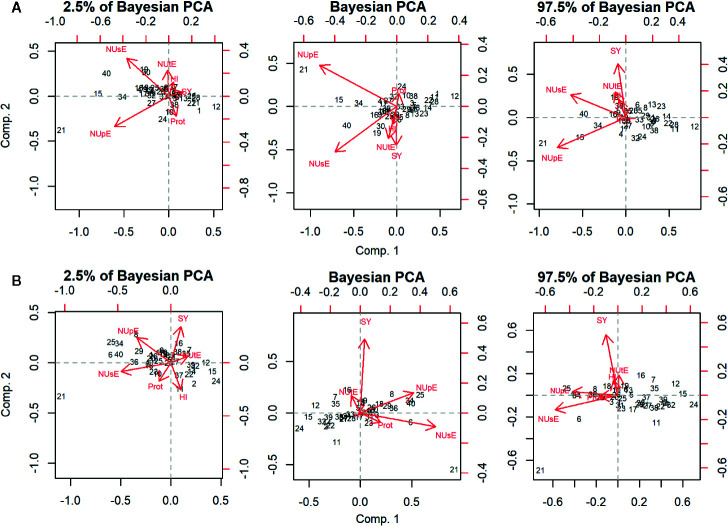
Principal component analysis (PCA) and the respective 95% highest posterior density (HPD) intervals of 40 *carioca* common bean cultivars under high **(A)** and low N **(B)** conditions evaluated for traits seed yield (SY), nitrogen use efficiency (NUsE), nitrogen uptake efficiency (NUpE), nitrogen utilization efficiency (NUtE), seed protein content (Prot), and harvest index (HI). A list of cultivars is presented in **Table 1**.

PCA graphics are shown in **Figure 1**. The first two principal components (Comp.1 and Comp.2) explained 76.1% (95% HPD = 60.1; 83.2%) and 14.3% (95% HPD = 6.1; 20.2%) of the total variation observed under high N condition. Under low N, Comp.1 and Comp.2 explained 74.2% (95% HPD = 57.4; 82.1%) and 15.2% (95% HPD = 8.0; 23.4%) of the total variation detected, respectively (**Supplementary Figure 2**). NUsE, NUpE, and SY were the traits that most contributed to discriminate cultivars under high and low N since they showed the highest absolute values for their eigenvectors in Comp.1 and Comp.2 (**Supplementary Figure 3**). The projection of NUtE and SY vectors showed the same direction in both N conditions. However, their vectors were projected in the opposite direction in relation to Prot, indicating a negative correlation of NUtE and SY to Prot. The IAC Alvorada cultivar was projected more distantly among all cultivars evaluated for both N conditions, probably because this cultivar showed the highest NUpE and NUsE means under high and low N conditions, respectively.

### Genetic Progress

Genetic progress estimates for the traits evaluated under high and low N are shown in **Table 6**. The significance of the linear regression model was observed only for SY in both N conditions since the estimates of the *β*_1_ parameter and their respective HPD intervals did not overlap the value of zero. Under high N, genetic progress estimate was 13.1 kg ha^−1^ (95% HPD = 9.3; 17.0 kg ha^−1^) or 0.53% year^−1^ (95% HPD = 0.39; 0.65%). Under low N condition there was a progress of 10.2 kg ha^−1^ (95% HPD = 6.1; 14.5 kg ha^−1^) or 0.48% year^−1^ (95% HPD = 0.31; 0.64%). There was no difference between the *a posteriori* slope estimates (Δ*β*_1_), indicating that the genetic progress of SY was the same in both N conditions.

**Table 6:** Genetic progress estimates and their respective 95% highest posterior density (HPD) intervals for traits seed yield (SY), nitrogen use efficiency (NUsE), nitrogen uptake efficiency (NUpE), nitrogen utilization efficiency (NUtE), seed protein content (Prot), and harvest index (HI).

Trait	Nitrogen	Parameter	Mean	95% HPD	Sig^1^	Δ*β*_1_^2^
SY (kg ha^−1^)	High	*β*_0_	2,495.6	(2,388.9; 2,609.1)		*ns*
		*β*_1_	13.1	(9.3; 17.0)	*	
		*σ*	517.6	(492.2; 544.5)		
	Low	*β*_0_	2,123.6	(1,995.8; 2,237.6)		
		*β*_1_	10.2	(6.1; 14.5)	*	
		*σ*	539.7	(512.5; 567.9)		
NUsE (g g^−1^)	High	*β*_0_	0.32	(0.21; 0.48)		*ns*
		*β*_1_	−0.0	(−0.0; 0.0)	*ns*	
		*σ*	0.1	(0.0; 0.1)		
	Low	*β*_0_	0.77	(0.56; 1.04)		
		*β*_1_	0.0	(−0.0; 0.0)	*ns*	
		*σ*	0.7	(0.6; 0.7)		
NUpE (mg g^−1^)	High	*β*_0_	0.12	(0.08; 0.16)		*ns*
		*β*_1_	−0.0	(−0.0; 0.0)	*ns*	
		*σ*	0.1	(0.0; 0.2)		
	Low	*β*_0_	0.28	(0.23; 0.33)		
		*β*_1_	0.0	(−0.0; 0.0)	*ns*	
		*σ*	0.2	(0.2; 0.3)		
NUtE (g mg^−1^)	High	*β*_0_	2.51	(2.26; 2.75)		*ns*
		*β*_1_	0.0	(−0.0; 0.0)	*ns*	
		*σ*	0.4	(0.3; 0.4)		
	Low	*β*_0_	2.68	(2.34; 2.86)		
		*β*_1_	0.0	(−0.0; 0.0)	*ns*	
		*σ*	0.6	(0.6; 0.7)		
Prot (%)	High	*β*_0_	20.21	(18.73; 20.98)		*ns*
		*β*_1_	0.0	(−0.0; 0.0)	*ns*	
		*σ*	2.4	(2.2; 2.5)		
	Low	*β*_0_	18.87	(17.71; 20.00)		
		*β*_1_	0.0	(−0.0; 0.0)	*ns*	
		*σ*	2.3	(2.2; 2.4)		
HI	High	*β*_0_	0.46	(0.39; 0.53)		*ns*
		*β*_1_	−0.0	(−0.0; 0.0)	*ns*	
		*σ*	0.1	(0.1; 0.1)		
	Low	*β*_0_	0.45	(0.38; 0.53)		
		*β*_1_	−0.0	(−0.0; 0.0)	*ns*	
		*σ*	0.1	(0.1; 0.1)		

^1^Angular coefficient (β_1_) of simple linear regression model were considered significant (*) when HPD intervals did not overlap the value of zero or not significant (ns) when there was overlap.

^2^Genetic progress differences (Δβ_1_) between high and low N conditions were considered significant (*) when there was no overlap of their HPD intervals or not significant (ns) when there was overlap.

### Adaptability and Stability

The *a* *posteriori* means of genotypic (*τ*) and environmental (*δ*) effects and their respective HPD intervals are shown in **Tables 7** and **8**, respectively. Overall mean (*µ*) of SY from 40 *carioca* common bean cultivars evaluated in eight contrasting N conditions was 2,155.71 kg ha^−1^ (95% HPD = 2141.99; 2169.54 kg ha^−1^). Twenty-one cultivars showed *a posteriori* positive means with HPD intervals that did not overlap the zero value (**Table 7**). Among these cultivars, the ones that stood out the most were: IPR Sabiá (376.61 kg ha^−1^; 95% HPD = 363.95; 389.91 kg ha^−1^), IPR Quero-quero (319.24 kg ha^−1^, 95% HPD = 306.23; 332.79 kg ha^−1^), IPR Bem-te-vi (288.76 kg ha^−1^; 95% HPD = 275.63; 302.05 kg ha^−1^), IAC Sintonia (274.92 kg ha^−1^; 95% HPD = 261.39; 287.95 kg ha^−1^), IPR Campos Gerais (235.44 kg ha^−1^; 95% HPD = 222.72; 24.06 kg ha^−1^), and BRS Notável (214.96 kg ha^−1^; 95% HPD = 201.86; 228.57 kg ha^−1^). It is worthwhile to mention that not all environments under high N conditions were considered as favorable environments since they did not show *a posteriori* positive means (**Table 8**).

**Table 7:** A *posteriori* mean of Bayesian additive main effects and multiplicative interaction (BAMMI) for seed yield (kg ha^−1^) of 40 *carioca* common bean cultivars in eight environments for the overall mean (µ) and genotypic effects (τ_i_) with their respective 95% highest posterior density (HPD).

Parameter	Mean	95% HPD	Adaptability	
			Wide	Specific
τ_1_ (Carioca)	−485.60	(−497.79; −472.52)	−	+
τ_2_ (IAPAR 14)	−204.48	(−218.16; −191.47)	−	+
τ_3_ (IAPAR 57)	−250.42	(–263.41; −237.25)	+	−
τ_4_ (IAPAR 72)	−246.32	(−259.48; −233.64)	−	+
τ_5_ (Pérola)	140.57	(128.04; 152.78)	−	−
τ_6_ (IAPAR 80)	−290.49	(−303.51; −277.76)	+	−
τ_7_ (IAPAR 81)	−79.03	(−92.43; −66.43)	−	+
τ_8_ (FTS Bonito)	−325.19	(−337.99; −312.06)	+	−
τ_9_ (ANFc 9)	−62.49	(−74.93; −49.74)	+	−
τ_10_ (Princesa)	−15.82	(−28.51; −3.09)	+	−
τ_11_ (IPR Juriti)	172.19	(158.62; 185.75)	+	−
τ_12_ (BRS Talismã)	−142.63	(−155.62; −130.35)	+	−
τ_13_ (BRS Pontal)	78.09	(64.79; 90.13)	−	−
τ_14_ (BRS Requinte)	−43.29	(−56.58; −30.79)	+	−
τ_15_ (IPR Saracura)	83.77	(70.78; 96.82)	−	−
τ_16_ (IPR Colibri)	−281.90	(−294.99; −269.02)	+	−
τ_17_ (BRS Horizonte)	−88.65	(−101.42; −75.18)	−	+
τ_18_ (BRS Pioneiro)	178.22	(165.65; 191.01)	+	−
τ_19_ (IPR Eldorado)	−20.17	(−33.10; −7.44)	−	+
τ_20_ (IPR 139)	166.18	(152.78; 179.52)	−	−
τ_21_ (IAC Alvorada)	−212.33	(−225.35; −199.64)	−	−
τ_22_ (IPR Tangará)	121.71	(107.89; 135.19)	+	−
τ_23_ (FTS 65)	128.49	(115.62; 141.29)	+	−
τ_24_ (BRS Estilo)	109.12	(95.57; 121.94)	−	−
τ_25_ (TAA Bola Cheia)	27.018	(14.21; 40.07)	−	+
τ_26_ (IPR Campos Gerais)	235.44	(222.72; 248.06)	+	−
τ_27_ (IPR Andorinha)	−20.40	(−33.16; −6.92)	−	−
τ_28_ (BRSMG Madrepérola)	127.08	(114.14; 139.64)	+	−
τ_29_ (BRSMG Ametista)	97.05	(83.99; 110.09)	+	−
τ_30_ (IPR Curió)	−177.35	(−190.66; −164.38)	−	−
τ_31_ (BRS Notável)	214.96	(201.86; 228.57)	−	−
τ_32_ (IAC Imperador)	−26.69	(−39.48; −13.22)	−	−
τ_33_ (TAA Gol)	−81.53	(−94.41; −67.87)	+	−
τ_34_ (IPR Maracanã)	−111.65	(−124.58; −99.19)	−	−
τ_35_ (TAA Dama)	99.10	(86.40; 112.77)	+	−
τ_36_ (IPR Quero-quero)	319.24	(306.23; 332.79)	+	−
τ_37_ (IPR Bem-te-vi)	288.76	(275.63; 302.05)	+	−
τ_38_ (IPR Celeiro)	−72.10	(−84.60; −58.96)	+	−
τ_39_ (IAC Sintonia)	274.92	(261.39; 287.95)	+	−
τ_40_ (IPR Sabiá)	376.61	(363.95; 389.91)	+	−
µ (overall mean)	2,155.71	(2,141.99; 2,169.54)		

**Table 8:** A *posteriori* mean of Bayesian additive main effects and multiplicative interaction (BAMMI) for seed yield (kg ha^−1^) of 40 *carioca* common bean cultivars in eight environments for general average (*µ*) and environment effect (*δ_j_*) with their respective 95% highest posterior density (HPD).

Parameter^1^	Mean	95% HPD	Environment
*δ*_1_ (LD17–HN)	232.05	(219.93; 244.79)	Favorable
*δ*_2_ (LD17–LN)	−303.95	(−316.03; −292.11)	Unfavorable
*δ*_3_ (PG17–HN)	580.23	(567.97; 592.32)	Favorable
*δ*_4_ (PG17–LN)	333.25	(321.17; 345.24)	Favorable
*δ*_5_ (PG18–HN)	215.59	(203.89; 227.68)	Favorable
*δ*_6_ (PG18–LN)	−86.96	(−99.84; −74.02)	Unfavorable
*δ*_7_ (STO18–HN)	−462.62	(−475.31; −450.28)	Unfavorable
*δ*_8_ (STO18–LN)	−507.59	(−520.14; −495.39)	Unfavorable
µ (overall mean)	2155.71	(2,141.99; 2,169.54)	

^1^LD, Londrina; PG, Ponta Grossa; STO, Santa Tereza do Oeste; 17, rainy season of 2017; 18, dry season of 2018; HN, high nitrogen; and LN, low nitrogen.

**Figure 2:**
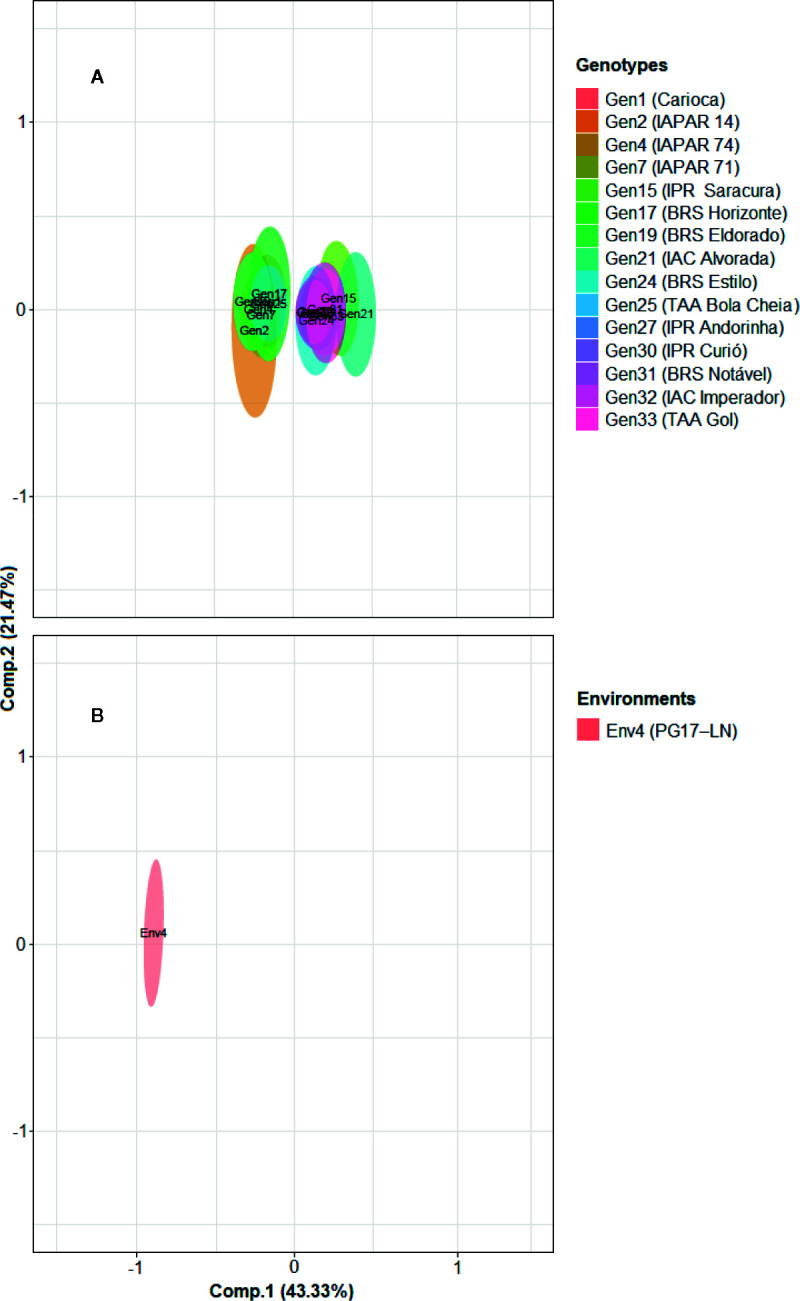
Genotype **(A)** and environment **(B)** scores with their respective 95% highest posterior density (HPD) intervals of 40 *carioca* common bean cultivars evaluated in eight contrasting environments. Cultivars and environment not plotted on the graphs showed HPD intervals overlapping with zero value in both principal components (Comp.1 and Comp.2).

Genotypic and environmental scores with their respective HPD intervals for the first two principal components (Comp.1 and Comp.2) are shown in **Figure 2**. Comp.1 and Comp.2 explained 43.33 and 21.47% of G × E interaction, respectively. The HPD intervals overlapping in the central point indicate the presence of genotypic or environmental stability. In addition, HPD intervals overlapping among genotypes or environments indicate similar responses among them. The genotypic scores showed that 25 cultivars have high stability since their HPD intervals overlapped with zero values on both axes (**Figure 2A**). The environmental scores allowed us to observe that PG17–LN was the environment which contributed more to G × E interaction since HPD interval did not overlap zero on the axis of Comp.1 (**Figure 2B**).

Comp.1 and Comp.2 coordinates allowed us to infer about the genotype specific adaptation to certain environments (**Figure 2**). Considering only genotypes and environments with significant contribution to G × E interaction, seven cultivars (Carioca, IAPAR 14, IAPAR 72, IAPAR 81, BRS Horizonte, IPR Eldorado, and TAA Bola Cheia) showed specific adaptation to environment PG17–LN. The cultivars that showed to be more adapted and stable were: IPR Sabiá, IPR Quero-quero, IPR Bem-te-vi, IAC Sintonia, and IPR Campos Gerais. These cultivars presented high SY mean values and high behavior predictability for different environmental conditions.

**Figure 3:**
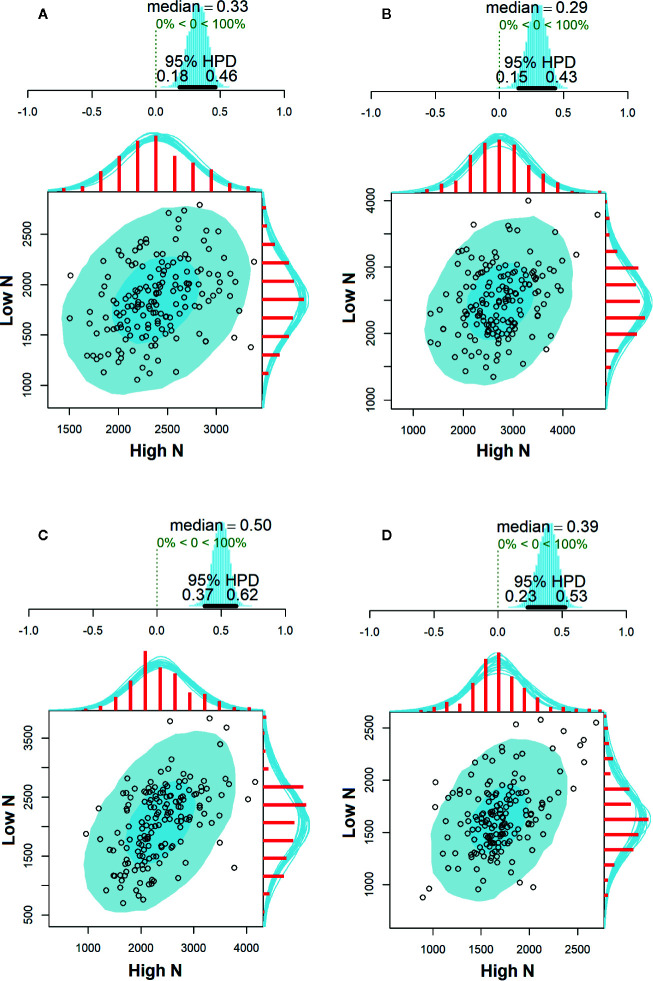
Correlation estimates of seed yield (kg ha^−1^) with their respective 95% highest posterior density (HPD) intervals between high and low N conditions evaluated in Londrina—2017 **(A)**, Ponta Grossa—2017 **(B)**, Ponta Grossa—2018 **(C)**, and Santa Tereza do Oeste—2018 **(D)**.

The environmental correlations of SY between high and low N conditions are shown in **Figure 3**. In general, correlation estimates were positive and significant in all evaluated environments since their HPD intervals did not overlap the zero. Moderate correlation estimates were observed in PG18 (*r* = 0.50, 95% HPD = 0.37; 0.62), while weak correlations were observed in STO18 (*r* = 0.39, 95% HPD = 0.23; 0.53), LD2017 (*r* = 0.33, 95% HPD = 0.18; 0.46), and PG2017 (*r* = 0.29, 95% HPD = 0.15; 0.43).

## Discussion

Our study was the first to quantify the genetic progress of *carioca* common beans in Brazil over the past 47 years under N contrasting conditions. In Brazil, these studies have mainly focused on the impact of breeding programs on plant architecture and yield components. For example, Barili et al. (2016) reported a change in modern cultivar architecture since there was a substitution of prostrate cultivars by erect growth habit. This change allowed mechanical harvesting with reduced losses, lower incidence of disease, and improved grain quality. In addition, the increase in common bean production has also been associated with genetic progress in the number of pods per plant (5.6% year^−1^), seeds per plant (4.5% year^−1^), mass of seeds (2.08% year^−1^), and tolerance to lodging (2.0% year^−1^) (de Faria et al., 2013; Barili et al., 2016). In common bean, there are still no studies quantifying the genetic progress for NUsE. On the other hand, an increase in NUsE has been reported in several other crops (Laidig et al., 2017; Stahl et al., 2017; Mueller et al., 2019).

### Interaction Between Factors and Influence of Nitrogen Fertilization

The best adjustment of the full model for all agronomic traits evaluated indicated that cultivars have a differential behavior based on the combination of N fertilization levels and environments in which the cultivars were submitted. The interaction between genotypes and environments is frequently observed in common bean breeding programs in Brazil (Barili et al., 2016; Pereira et al., 2018) and worldwide (Bruno et al., 2017; Caproni et al., 2018).

SY increased under high N top-dressing (17.51%), corroborating a previous study that reported N as the macro-element that most influences SY in common bean crop (Barros et al., 2018). Similar results were also observed for 16 Brazilian *carioca* common bean cultivars which observed an increase of approximately 21.71% in plants under high N condition (Leal et al., 2019).

The majority of the NUsE and NUpE evaluated in *carioca* common bean cultivars in this study were higher under low N compared to the high N condition. Studies have already been reported that plants improve their NUsE under low N availability, especially because under high N conditions this element can be lost by leaching, denitrification, and volatilization processes (Shibata et al., 2017).

Several studies also reported the influence of environmental factors on Prot in common beans (Pereira et al., 2017; Fidelis et al., 2019). A study using 40 common bean genotypes (*carioca* and black types) observed 18.33 and 19.36% of Prot in common beans cultivated in low and high N condition, respectively, where 17 genotypes did not show an increase in Prot in the presence of N top-dressing (Fidelis et al., 2019). In the present study, although the overall mean of Prot was not significantly altered by N top-dressing, seven cultivars (BRS Requinte, BRS Horizonte, IAC Alvorada, FTS 65, IPR Campos Gerais, BRS Notável, and IPR Quero-quero) showed an increase in Prot under the high N condition.

We did not observe significant differences for HI values between both N conditions. However, some studies observed an increase of HI through N top-dressing fertilization (Fageria and Baligar, 2005; Fageria et al., 2014). HI is a measure of biomass partitioning, which indicates the fraction of total above-ground biomass allocated in the seeds and is considered to be one of the most determinant traits of common bean tolerance to several abiotic stresses (Assefa et al., 2015; Amanuel et al., 2018). In general, the genotypes that are more tolerant to abiotic stresses have a higher HI value, that is, a greater ability to partition photo-assimilates from vegetative organs to seeds (Assefa et al., 2015). In common bean, HI values have varied between 0.3 and 0.6, being considered a complex trait and strongly influenced by environment (Pinto Júnior et al., 2018). According to Beebe et al. (2008), the increase in HI is a key strategy in the improvement of beans, mainly under abiotic stress conditions.

### Agronomic Traits Correlations

NUtE was the only component of NUsE to present a positive correlation with SY in both N conditions, indicating that this trait is the main component of NUsE in common bean crop, especially when the breeding program aims to increase SY. Several metabolic and physiological mechanisms are associated with NUtE in plants, including greater photosynthetic efficiency per unit of N, improved partition of carbohydrates, storage, and N remobilization through senescent tissues (Han et al., 2015). NUtE increase is often associated to the stay-green character since genotypes with this characteristic maintain leaves photosynthetically active for a longer period (Zhang et al., 2020). HI has also been associated with NUtE since its increase contributes to a more efficient production of seed biomass by total biomass accumulated in plants (Han et al., 2015). In this study, we also observed a positive correlation between HI and NUtE in both N conditions.

We observed a negative correlation between NUtE and NUpE under low N condition, similar to what has been previously described in maize (Gallais and Hirel, 2004). These authors reported that NUtE is related to protein degradation in senescent leaves, and they assumed that the use of N occurs mainly when absorption is reduced or interrupted during stresses and/or natural senescence. In addition, the genetic variability of NUsE is a function of the existing variability of NUpE in high N condition, while in low availability it is a function of NUtE. Our results partially corroborated this study since we observed a genetic variability in NUpE with a greater contribution of NUsE in both N conditions.

The observed negative correlations between SY and Prot may be related to altered patterns of carbon and N metabolism, and this result is corroborated by different studies carried out in common bean (Razvi et al., 2018) and other crops, such as, oilseed rape (Stahl et al., 2017) and wheat (Thorwarth et al., 2019). Several studies investigated the genetic basis of this negative correlation and showed that pleiotropic effects, environmental conditions, and management techniques can influence the relationship between these variables (Rozbicki et al., 2015; Thorwarth et al., 2019). Another study reported genetic correlations between Prot and SY (*r_g_* = 0.51; *p* < 0.01) similar to those obtained in the present study using 140 common bean recombinant inbred lines (RIL) under contrasting N condition (Farid et al., 2017).

### Genetic Progress

Our genetic progress related to SY was similar under low (0.48%, 95% HPD = 0.31; 0.64%) and high N (0.53%, 95% HPD = 0.39; 0.65%), indicating that modern cultivars do not demand more N fertilization to be more productive than cultivars released earlier. Around ten years are needed to develop a new common bean cultivar (Moreira et al., 2010). As the number of selected lines is reduced, the range of environments in which they are tested is wider. Among all these trials, moderate N stresses surely occur unintentionally. Thus, the selection process may already mix high and low N environments explaining in part the similar genetic progress observed in the present study under both N conditions.

Genetic progress estimates for SY using Brazilian *carioca* and black common beans cultivars have already been reported (Chiorato et al., 2010; de Faria et al., 2013; Barili et al., 2016), but not under low N condition. For example, de Faria et al. (2013) assessing lines and cultivars representative of 22 years of the Embrapa breeding program reported progress of 0.72% year^−1^ (17.3 kg ha^−1^). In the IAC bean breeding program, genetic progress was 1.07% year^–1^ (13.17 kg ha^–1^) between 1989 and 1996. Differences between germplasm, environments, methodologies, and statistical methods are the main reasons for the differences observed among studies (de Faria et al., 2018).

We did not detect a significant difference in Prot content among modern and old cultivars. Our hypothesis is that selection on Prot may only result in the elimination of low Prot lines and not in increasing Prot, likewise reported in wheat (Cormier et al., 2013) and oilseed rape (Stahl et al., 2017) crops. Common bean breeding program objectives were clearly to increase SY, and the concern and interest in biofortification is recent. Since there is a negative correlation between SY and Prot, an alternative could be to improve protein composition to increase the levels of essential amino acids (mainly methionine, cysteine, and tryptophan) and/or decrease protein digestibility inhibitors (Rezende et al., 2017).

Although there is wide genetic variability observed for NUsE, NUtE, and NUpE in this study, no genetic progress was detected for these traits. Possibly the common bean breeding programs are not focusing in the improvement of NUsE since common beans are able to fix N_2_ by symbiosis. However, NUsE genetic progress and their components have been reported in other crops that do not have this N_2_ fixation ability, such as, wheat (Laidig et al., 2017), maize (Mueller et al., 2019), and oilseed rape (Stahl et al., 2017). In addition, the evaluation and selection for NUsE is considered an extremely expensive process and may not be implemented in breeding programs soon. High-throughput phenotyping methods are currently being developed (Neilson et al., 2015; Kefauver et al., 2017) and can become an important tool in the improvement of NUtE and/or NUpE in addition to molecular selection on genes or quantitative trait loci (QTL) (Cormier et al., 2013).

In our study, the genetic progress of HI was absent and indicated that superior genotypes selection was not performed focusing on this agronomic trait. However, the genetic progress of HI has been historically reported in several other crops (Cormier et al., 2013; Mueller et al., 2019; Todeschini et al., 2019). In a meta-analysis including eleven different crops, the authors reported a positive relationship between SY and HI in cereal (maize, wheat, barley, and oat), oilseed (soybean, canola, and flax), and pulse crops (pea, chickpea, and lentil), with the exception of potato crops only (Fan et al., 2017). The same authors affirmed that this positive linear relationship indicates that SY improvement was achieved in part through HI, and that plant breeders should focus greater attention on this agronomic trait to develop new cultivars.

### Adaptability and Stability

The development of genotypes with environment adaptive plasticity, good stability, and high SY mean is one way to mitigate G × E interaction effects (Resende et al., 2019). Statistical methods to study adaptability and stability have been developed and widely used by plant breeding programs (Nascimento et al., 2020). The BAMMI method stands out for its power to explain G × E interaction compared to other methods (Teodoro et al., 2019). Another advantage of the BAMMI model is the presence of HPD intervals for genotypic and environment scores (Oliveira et al., 2015). These credibility intervals lead to greater precision to infer genotypic and environment stability by eliminating subjective mean scores in relation to the proximity to the central point of the biplot (coordinates 0 and 0).

In the present study, the large number of cultivars considered stable is due to the selection for stability in several environment conditions by their respective breeding programs. In addition, there was no relationship between stability and release year since both modern and old cultivars showed high stability. The modern cultivars IPR Sabiá, IPR Quero-quero, IPR Bem-te-vi, IAC Sintonia, and IPR Campos Gerais were the most adapted and stable in the present study. These results confirm that common bean breeding programs are developing cultivars that combine SY and stability for different environmental conditions.

We observed that not all environments under high N condition were considered favorable environments by BAMMI. In addition, the positive correlations between high and low N for all environments explain the similar genetic progress among them for SY. These results indicated that even though common bean breeding programs have made selection under N fertilization, modern cultivars do not require high N levels to achieve their genetic potential. Similar results were already reported in maize (Haegele et al., 2013) and wheat (Cormier et al., 2013).

## Conclusion

Our study is the first to quantify the genetic progress for SY and NUsE-related traits in Brazilian *carioca* common bean cultivars launched from 1970 to 2017 under N contrasting conditions. Among the traits evaluated, there was genetic progress only for SY under high and low N in top-dressing. The similar genetic progress in both N conditions rejected our hypothesis that modern cultivars are more N-dependent to reach their productive potential. Our results indicate that the challenges for common bean breeders for the coming years will be to improve the NUsE of new cultivars in order to reduce the dependence on N fertilizers as well as to increase the levels and/or quality of Prot in the seeds.

## Data Availability Statement

The raw data supporting the conclusions of this article will be made available by the authors, without undue reservation.

## Author Contribuitions

DZ, IM, VM-C, and LG conceived and designed the experiments. DZ, IM, and JD conducted the experiments and collected the data. DZ and GF performed the statistical analyses. DZ wrote the original draft. S-IS, VM-C, JN, GF, CS, and LG read and edited the manuscript. All authors contributed to the article and approved the submitted version.

## Conflict of Interest

The authors declare that the research was conducted in the absence of any commercial or financial relationships that could be construed as a potential conflict of interest.
