# Supplementary material for: Genetic Progress of Seed Yield and Nitrogen Use Efficiency of Brazilian carioca Common Bean Cultivars Using Bayesian Approaches
Source: Front Plant Sci. 2020 Aug 5;11:1168. doi: 10.3389/fpls.2020.01168 (PMC7419646; doi:10.3389/fpls.2020.01168)
Supplement: Supplementary file 1 [file Table_1.docx]

**Supplementary Tables**

**Supplementary Table 1.** Genealogy of 40 Brazilian *carioca* common bean cultivars.

| Cultivar | Genealogy |
| --- | --- |
| IAPAR 14 | – |
| IAPAR 57 | – |
| IAPAR 72 | – |
| IAPAR 80 | BAT 93 / 2 / Carioca Sel. 99 / Great Northern Nebraska 1 Sel. 27 / 3 / Sel. Aroana / 4 / A176 / A259 |
| IAPAR 81 | A248 / EMP117 / 4 / BAT 93 / 2 / Carioca Sel. 99 / Great Northern Nebraska 1 # 27 / 3 / Sel. Aroana |
| IPR 139 | – |
| IPR Tangará | LP 95–92 / Pérola |
| IPR Juriti | BAT 93 / 2 / Carioca Sel. 99 / Great Northern Nebraska 1 Sel. 27 / 3 / Sel. Aroana / 4 / A176 / A259 / 5 / ll 130 / XAN87 |
| IPR Quero-quero | LP02-21 / LP02-22 |
| IPR Saracura | LPSPI 93–08 / IAPAR 31 |
| IPR Colibri | Selection in ‘Carioca Pitoco’ landrace |
| IPR Eldorado | RM8454–21–1–cm / IAPAR 14 |
| IPR Campos Gerais | IAPAR 80 / Campeão 2 |
| IPR Curió | IAPAR 81 / Carioca 1070 line |
| IPR Andorinha | SEL37–20 / IPR Colibri |
| IPR Bem-te-vi | BAT 93 / 2 / Carioca sel. 99/ Great Northern Nebraska 1 Sel. 27 / 3 / Sel. Aroana / IAPAR 31 / 4 / Campeão 1 |
| IPR Celeiro | Porrillo Sintetico / MD821 // RM8454-8-4-cm / Carnaval |
| IPR Sabiá | – |
| Pérola | Selection within Aporé cultivar |
| BRS Pontal | BZ3836 // FEB 166/AN 910523 |
| BRS Requinte | Carioca MG // POT 94 / AN 910523 |
| BRSMG Talismã | Recurrent selection including BAT 477, IAPAR 14, FT 84-29, Jalo EEP, A 252, A 77, Ojo de Liebre, ESAL 645, Pintado and Carioca |
| BRS Estilo | EMP250 /4/ A769 /// A429 / XAN 252 // V8025 / Pinto VI 114 |
| BRS Notável | A 769 / 4 / A 774 /// A 429 / XAN 252 // V 8025 / G 4449 /// WAF 2 /A55 // GN 31 / XAN 170 |
| BRSMG Madrepérola | AN 512666–0 / AN 730031 |
| BRS Ametista | PR9115957 / LR720982CP |
| BRSMG Horizonte | EMP 250 / 4 / A769 /// XAN 252 / Pinto VI 114 |
| BRSMG Pioneiro | Rudá / Ouro Negro |
| IAC Alvorada | IAC Carioca Pyatã / A686 / 2 / IAC Maravilha / G2338 / 3 / IAC Maravilha / And277 / 4 / L317-1 |
| IAC Imperador | IAC Carioca Eté / Carioca Precoce / 2 / IAC Carioca Eté / 3 / Feijão 60 days |
| FTS Bonito | – |
| Carioca | Farmers selection |
| ANFc 9 | – |
| Princesa | – |
| FTS 65 | – |
| TAA Bola Cheia | WB-SR9903 / IAPAR 31 |
| TAA Gol | – |
| IPR Maracanã | MD 632 / IAPAR BAC 32 |
| IAC Sintonia | IAC Alvorada / Pérola |
| TAA Dama | – |

**Supplementary Table 2.** Mean values of marginal *a posteriori* effects with their respective 95% highest posterior density (HPD) for seed yield (SY) and protein content (Prot) evaluated in 40 *carioca* common bean cultivars under high and low N condition.

| Cultivar^1^ | SY (kg ha^–1^) | |  | Prot (%) | |
| --- | --- | --- | --- | --- | --- |
|  | High N | Low N |  | High N | Low N |
| 1 | 2134 (2058; 2326) | 2021 (1787; 2067) |  | 19.2 (18.8; 20.8) | 18.6 (16.6; 19.9) |
| 2 | 2264 (2190; 2337) | 2063 (1957; 2218) |  | 20.3 (18.7; 21.1) | 17.8 (16.4; 18.9) |
| 3 | 2492 (2303; 2537) | 1985 (1840; 2056) |  | 19.8 (18.0; 21.1) | 19.2 (18.6; 20.3) |
| 4 | 2271 (2213; 2484) | 2111 (1968; 2165) |  | 21.6 (19.7; 22.2) | 18.9 (17.5; 20.1) |
| 5 | 2790 (2653; 2846) | 2254 (2088; 2341) |  | 18.1 (17.2; 19.6) | 18.6 (17.3; 20.0) |
| 6 | 2109 (2029; 2266) | 1900 (1827; 2081) |  | 19.3 (17.9; 20.8) | 18.5 (17.5; 19.5) |
| 7 | 2567 (2407; 2747) | 2145 (1970; 2303) |  | 19.2 (17.6; 20.5) | 19.4 (19.0; 20.1) |
| 8 | 2397 (2281; 2462) | 2118 (1938; 2162) |  | 19.5 (18.1; 21.2) | 18.7 (17.3; 19.6) |
| 9 | 2549 (2473; 2654) | 2176 (1921; 2220) |  | 19.7 (18.0; 21.5) | 19.1 (18.1; 19.5) |
| 10 | 2422 (2352; 2547) | 2140 (2051; 2306) |  | 19.2 (19.0; 20.9) | 19.5 (18.1; 20.6) |
| 11 | 2712 (2563; 2767) | 2362 (2143; 2404) |  | 19.5 (18.5; 20.1) | 20.5 (19.2; 21.9) |
| 12 | 2455 (2324; 2535) | 1900 (1785; 1991) |  | 20.5 (19.3; 21.7) | 20.3 (18.9; 21.4) |
| 13 | 2675 (2546; 2759) | 2037 (1966; 2238) |  | 19.9 (18.6; 20.6) | 19.0 (18.6; 19.6) |
| 14 | 2549 (2439; 2735) | 2267 (1983; 2342) |  | 20.5 (20.0; 21.8) | 18.5 (17.4; 19.6) |
| 15 | 2360 (2289; 2568) | 2196 (2023; 2244) |  | 18.6 (18.1; 19.9) | 18.1 (17.4; 18.6) |
| 16 | 2274 (2227; 2395) | 1890 (1779; 1956) |  | 20.2 (18.6; 21.5) | 18.6 (17.4; 19.9) |
| 17 | 2479 (2427; 2619) | 1970 (1896; 2116) |  | 22.1 (20.8; 23.3) | 19.1 (18.3; 20.4) |
| 18 | 2450 (2318; 2662) | 2076 (1987; 2169) |  | 19.7 (17.8; 20.9) | 18.8 (17.6; 20.2) |
| 19 | 2450 (2319; 2511) | 2239 (2157; 2354) |  | 19.4 (18.6; 20.7) | 18.1 (16.6; 20.3) |
| 20 | 2680 (2598; 2783) | 2097 (2031; 2282) |  | 19.2 (18.6; 20.0) | 18.7 (17.5; 19.8) |
| 21 | 2424 (2345; 2553) | 1927 (1851; 2086) |  | 21.3 (19.2; 22.6) | 17.9 (16.2; 19.1) |
| 22 | 2686 (2528; 2789) | 2304 (2176; 2376) |  | 20.3 (19.0; 21.6) | 20.4 (19.2; 21.5) |
| 23 | 2760 (2551; 2811) | 2175 (2094; 2271) |  | 22.2 (21.0; 23.2) | 18.4 (18.0; 18.9) |
| 24 | 2690 (2606; 2815) | 2210 (2080; 2265) |  | 22.9 (19.3; 20.0) | 19.0 (16.1; 19.9) |
| 25 | 2516 (2391; 2574) | 2185 (2124; 2294) |  | 19.7 (18.8; 20.0) | 19.4 (18.1; 20.4) |
| 26 | 2870 (2665; 2930) | 2172 (2047; 2343) |  | 20.3 (19.6; 20.8) | 17.7 (16.5; 18.8) |
| 27 | 2362 (2291; 2492) | 1999 (1970; 2128) |  | 21.0 (19.9; 21.2) | 21.1 (20.0; 22.4) |
| 28 | 2639 (2541; 2688) | 2048 (1949; 2197) |  | 20.1 (19.4; 20.8) | 19.8 (18.1; 21.1) |
| 29 | 2482 (2451; 2648) | 2213 (2104; 2253) |  | 19.9 (18.5; 21.7) | 19.3 (18.6; 20.2) |
| 30 | 2604 (2430; 2647) | 1913 (1871; 2115) |  | 20.4 (18.7; 21.5) | 19.3 (18.0; 21.1) |
| 31 | 2551 (2429; 2648) | 2131 (1949; 2171) |  | 21.6 (20.0; 22.3) | 18.7 (17.4; 19.8) |
| 32 | 2463 (2416; 2607) | 2059 (1941; 2271) |  | 19.8 (18.2; 21.1) | 19.8 (18.5; 21.4) |
| 33 | 2063 (1965; 2287) | 2233 (2034; 2332) |  | 18.9 (17.2; 20.5) | 18.3 (17.6; 19.2) |
| 34 | 2424 (2317; 2474) | 1870 (1818; 2122) |  | 20.1 (18.7; 21.1) | 18.5 (17.7; 19.1) |
| 35 | 2423 (2387; 2560) | 2146 (2084; 2394) |  | 18.2 (17.6; 18.8) | 18.2 (16.8; 19.5) |
| 36 | 2607 (2544; 2717) | 2380 (2200; 2504) |  | 19.2 (18.3; 20.4) | 17.2 (16.8; 18.1) |
| 37 | 2764 (2551; 2835) | 2361 (2154; 2403) |  | 19.8 (18.2; 21.0) | 18.9 (17.3; 20.1) |
| 38 | 2470 (2353; 2549) | 2102 (1999; 2180) |  | 19.2 (18.9; 20.3) | 18.8 (17.4; 20.0) |
| 39 | 2518 (2433; 2596) | 2132 (2086; 2292) |  | 19.3 (18.6; 20.3) | 19.4 (18.2; 20.4) |
| 40 | 2412 (2337; 2584) | 2422 (2185; 2475) |  | 19.6 (18.5; 20.1) | 17.9 (16.8; 19.1) |

^1^List of cultivars is presented in Table 1.

**Supplementary Table 3.** Mean values of marginal *a posteriori* effects with their respective 95% highest posterior density (HPD) for harvest index (HI) and nitrogen use efficiency (NUsE) evaluated in 40 *carioca* common bean cultivars under high and low N condition.

| Cultivar^1^ | HI | |  | NUsE (g g^–1^) | |
| --- | --- | --- | --- | --- | --- |
|  | High N | Low N |  | High N | Low N |
| 1 | 0.46 (0.37; 0.51) | 0.45 (0.36; 0.45) |  | 0.32 (0.19; 0.46) | 0.55 (0.37; 0.73) |
| 2 | 0.45 (0.41; 0.50) | 0.54 (0.50; 0.68) |  | 0.23 (0.12; 0.30) | 0.76 (0.57; 1.00) |
| 3 | 0.50 (0.45; 0.55) | 0.51 (0.46; 0.55) |  | 0.33 (0.22; 0.48) | 0.58 (0.44; 0.77) |
| 4 | 0.48 (0.38; 0.58) | 0.47 (0.38; 0.49) |  | 0.29 (0.18; 0.46) | 0.85 (0.57; 1.22) |
| 5 | 0.48 (0.38; 0.58) | 0.47 (0.38; 0.51) |  | 0.38 (0.23; 0.60) | 0.75 (0.50; 1.08) |
| 6 | 0.50 (0.45; 0.53) | 0.44 (0.40; 0.47) |  | 0.35 (0.24; 0.49) | 1.24 (0.91; 1.48) |
| 7 | 0.46 (0.41; 0.55) | 0.44 (0.40; 0.51) |  | 0.28 (0.19; 0.44) | 0.72 (0.54; 1.04) |
| 8 | 0.47 (0.39; 0.53) | 0.46 (0.39; 0.51) |  | 0.31 (0.20; 0.46) | 0.75 (0.54; 1.03) |
| 9 | 0.46 (0.41; 0.55) | 0.43 (0.39; 0.47) |  | 0.31 (0.21; 0.49) | 0.69 (0.52; 0.99) |
| 10 | 0.47 (0.42; 0.52) | 0.46 (0.41; 0.54) |  | 0.35 (0.24; 0.51) | 1.14 (0.91; 1.44) |
| 11 | 0.46 (0.41; 0.55) | 0.44 (0.40; 0.51) |  | 0.29 (0.20; 0.46) | 0.81 (0.61; 1.17) |
| 12 | 0.47 (0.33; 0.56) | 0.49 (0.34; 0.58) |  | 0.25 (0.13; 0.40) | 0.59 (0.35; 0.85) |
| 13 | 0.47 (0.42; 0.49) | 0.46 (0.41; 0.53) |  | 0.32 (0.22; 0.44) | 0.80 (0.60; 1.01) |
| 14 | 0.43 (0.39; 0.52) | 0.39 (0.34; 0.43) |  | 0.28 (0.19; 0.44) | 0.67 (0.51; 0.96) |
| 15 | 0.49 (0.44; 0.54) | 0.63 (0.57; 0.71) |  | 0.45 (0.31; 0.65) | 0.79 (0.60; 1.04) |
| 16 | 0.48 (0.38; 0.58) | 0.50 (0.40; 0.60) |  | 0.24 (0.15; 0.38) | 0.65 (0.44; 0.94) |
| 17 | 0.46 (0.37; 0.55) | 0.43 (0.34; 0.46) |  | 0.33 (0.20; 0.52) | 0.67 (0.45; 0.96) |
| 18 | 0.47 (0.42; 0.49) | 0.46 (0.41; 0.51) |  | 0.30 (0.20; 0.42) | 0.83 (0.63; 1.05) |
| 19 | 0.46 (0.41; 0.48) | 0.46 (0.41; 0.52) |  | 0.20 (0.14; 0.28) | 0.54 (0.41; 0.68) |
| 20 | 0.46 (0.32; 0.55) | 0.44 (0.31; 0.55) |  | 0.32 (0.17; 0.51) | 0.74 (0.44; 1.07) |
| 21 | 0.47 (0.42; 0.56) | 0.43 (0.39; 0.51) |  | 0.69 (0.57; 0.92) | 1.85 (1.60; 2.26) |
| 22 | 0.45 (0.36; 0.50) | 0.46 (0.37; 0.55) |  | 0.34 (0.21; 0.49) | 0.62 (0.42; 0.82) |
| 23 | 0.46 (0.41; 0.51) | 0.46 (0.41; 0.55) |  | 0.25 (0.17; 0.36) | 0.60 (0.45; 0.79) |
| 24 | 0.48 (0.38; 0.53) | 0.48 (0.38; 0.58) |  | 0.25 (0.15; 0.36) | 0.78 (0.52; 1.03) |
| 25 | 0.45 (0.36; 0.54) | 0.46 (0.37; 0.51) |  | 0.34 (0.21; 0.54) | 0.92 (0.62; 1.32) |
| 26 | 0.47 (0.42; 0.56) | 0.44 (0.40; 0.52) |  | 0.27 (0.18; 0.43) | 0.72 (0.54; 1.04) |
| 27 | 0.45 (0.36; 0.50) | 0.50 (0.40; 0.62) |  | 0.23 (0.14; 0.33) | 0.51 (0.34; 0.67) |
| 28 | 0.47 (0.42; 0.56) | 0.47 (0.42; 0.54) |  | 0.34 (0.23; 0.49) | 0.76 (0.57; 1.00) |
| 29 | 0.47 (0.42; 0.52) | 0.46 (0.41; 0.51) |  | 0.32 (0.22; 0.46) | 0.79 (0.60; 1.04) |
| 30 | 0.49 (0.34; 0.64) | 0.47 (0.33; 0.55) |  | 0.25 (0.13; 0.43) | 0.59 (0.35; 0.92) |
| 31 | 0.42 (0.38; 0.50) | 0.47 (0.42; 0.58) |  | 0.28 (0.19; 0.44) | 0.48 (0.36; 0.69) |
| 32 | 0.47 (0.33; 0.56) | 0.46 (0.32; 0.53) |  | 0.29 (0.15; 0.46) | 0.77 (0.45; 1.11) |
| 33 | 0.36 (0.31; 0.41) | 0.50 (0.45; 0.67) |  | 0.29 (0.20; 0.40) | 0.66 (0.50; 0.83) |
| 34 | 0.49 (0.39; 0.54) | 0.50 (0.40; 0.65) |  | 0.67 (0.42; 0.84) | 1.27 (0.95; 1.58) |
| 35 | 0.44 (0.40; 0.53) | 0.39 (0.35; 0.42) |  | 0.28 (0.19; 0.44) | 0.62 (0.47; 0.89) |
| 36 | 0.47 (0.42; 0.52) | 0.46 (0.41; 0.50) |  | 0.35 (0.24; 0.51) | 1.10 (0.95; 1.41) |
| 37 | 0.47 (0.42; 0.56) | 0.45 (0.41; 0.50) |  | 0.37 (0.25; 0.59) | 0.65 (0.49; 0.94) |
| 38 | 0.43 (0.39; 0.47) | 0.40 (0.36; 0.42) |  | 0.35 (0.24; 0.51) | 0.73 (0.55; 0.96) |
| 39 | 0.47 (0.33; 0.54) | 0.44 (0.31; 0.47) |  | 0.34 (0.18; 0.52) | 0.61 (0.36; 0.84) |
| 40 | 0.48 (0.43; 0.53) | 0.50 (0.45; 0.60) |  | 0.36 (0.24; 0.52) | 0.84 (0.64; 1.11) |

^1^List of cultivars is presented in Table 1.

**Supplementary Table 4.** Mean values of marginal a posteriori effects with their respective 95% highest posterior density (HPD) for nitrogen uptake efficiency (NUpE) and nitrogen utilization efficiency (NUtE) evaluated in 40 *carioca* common bean cultivars under high and low N condition.

| Cultivar^1^ | NUpE (mg g^–1^) | |  | NUtE (g mg^–1^) | |
| --- | --- | --- | --- | --- | --- |
|  | High N | Low N |  | High N | low N |
| 1 | 2.52 (2.02; 2.77) | 2.38 (1.90; 2.62) |  | 2.52 (2.29; 2.77) | 2.19 (2.02; 2.41) |
| 2 | 2.49 (2.24; 2.74) | 2.60 (2.34; 2.86) |  | 2.52 (2.28; 2.70) | 2.65 (2.57; 2.83) |
| 3 | 2.55 (2.30; 2.81) | 2.65 (2.39; 2.92) |  | 2.56 (2.33; 2.83) | 2.67 (2.42; 2.80) |
| 4 | 2.46 (1.97; 2.95) | 2.62 (2.10; 3.14) |  | 2.37 (2.11; 2.58) | 2.51 (2.33; 2.72) |
| 5 | 3.15 (2.48; 3.62) | 2.82 (2.30; 3.34) |  | 2.90 (2.77; 3.05) | 2.65 (2.49; 2.88) |
| 6 | 2.53 (2.28; 2.66) | 2.63 (2.37; 2.76) |  | 2.53 (2.37; 2.70) | 2.73 (2.52; 3.02) |
| 7 | 2.47 (2.22; 2.96) | 2.54 (2.29; 3.05) |  | 2.51 (2.21; 2.78) | 2.55 (2.34; 2.69) |
| 8 | 2.01 (1.71; 2.27) | 2.79 (2.40; 2.97) |  | 2.50 (2.22; 2.64) | 2.37 (2.17; 2.52) |
| 9 | 2.49 (2.24; 2.99) | 2.41 (2.17; 2.89) |  | 2.47 (2.29; 2.52) | 2.54 (2.39; 2.71) |
| 10 | 2.56 (2.30; 2.82) | 2.56 (2.30; 2.82) |  | 2.53 (2.28; 2.73) | 2.56 (2.04; 3.03) |
| 11 | 2.73 (2.46; 3.28) | 2.57 (2.31; 3.08) |  | 2.71 (2.62; 2.91) | 2.54 (2.20; 3.02) |
| 12 | 2.10 (1.38; 2.38) | 2.09 (1.37; 2.57) |  | 2.42 (2.30; 2.55) | 2.56 (2.33; 2.87) |
| 13 | 2.57 (2.31; 2.70) | 2.66 (2.39; 2.79) |  | 2.54 (2.11; 3.05) | 2.81 (2.48; 3.17) |
| 14 | 2.31 (2.08; 2.77) | 2.45 (2.21; 2.94) |  | 2.27 (2.18; 2.37) | 2.42 (2.27; 2.64) |
| 15 | 2.74 (2.47; 3.01) | 2.88 (2.59; 3.17) |  | 2.73 (2.58; 2.80) | 3.01 (2.64; 3.34) |
| 16 | 2.40 (1.92; 2.88) | 2.46 (1.97; 2.95) |  | 2.43 (1.94; 2.86) | 2.25 (1.77; 2.86) |
| 17 | 2.35 (1.88; 2.82) | 2.49 (1.99; 2.99) |  | 2.29 (1.94; 2.47) | 2.53 (2.39; 2.70) |
| 18 | 2.46 (2.21; 2.58) | 2.59 (2.33; 2.72) |  | 2.49 (2.36; 2.58) | 2.46 (2.13; 2.60) |
| 19 | 2.36 (2.12; 2.48) | 2.54 (2.29; 2.67) |  | 2.41 (2.23; 2.53) | 2.36 (2.15; 2.53) |
| 20 | 2.60 (1.82; 3.12) | 2.64 (1.85; 3.17) |  | 2.62 (2.55; 2.77) | 2.63 (2.21; 3.09) |
| 21 | 3.52 (3.27; 4.02) | 3.74 (3.37; 4.49) |  | 2.45 (1.99; 2.99) | 4.10 (3.90; 4.34) |
| 22 | 2.46 (1.97; 2.71) | 2.39 (1.91; 2.63) |  | 2.33 (2.27; 2.48) | 2.25 (2.12; 2.33) |
| 23 | 2.24 (2.02; 2.46) | 2.39 (2.15; 2.63) |  | 2.26 (2.13; 2.39) | 2.32 (2.29; 2.40) |
| 24 | 2.58 (2.06; 2.84) | 2.71 (2.17; 2.98) |  | 2.53 (2.16; 2.93) | 3.00 (2.73; 3.22) |
| 25 | 2.46 (2.27; 2.65) | 3.23 (2.84; 3.72) |  | 2.35 (2.22; 2.48) | 3.07 (2.89; 3.25) |
| 26 | 2.47 (2.22; 2.96) | 2.71 (2.44; 3.25) |  | 2.49 (2.28; 2.65) | 2.67 (2.15; 3.02) |
| 27 | 2.27 (1.82; 2.50) | 2.26 (1.81; 2.49) |  | 2.21 (1.95; 2.30) | 2.08 (1.63; 2.58) |
| 28 | 2.59 (2.33; 2.85) | 2.57 (2.31; 2.83) |  | 2.57 (2.16; 3.09) | 2.49 (2.36; 2.57) |
| 29 | 2.57 (2.31; 2.83) | 2.59 (2.33; 2.85) |  | 2.68 (2.55; 2.98) | 2.83 (2.67; 3.04) |
| 30 | 2.52 (1.76; 3.28) | 2.47 (1.73; 3.21) |  | 2.52 (1.82; 3.00) | 2.28 (1.76; 2.73) |
| 31 | 2.20 (1.98; 2.64) | 2.48 (2.23; 2.98) |  | 2.10 (1.93; 2.23) | 2.48 (2.35; 2.52) |
| 32 | 2.46 (1.72; 2.95) | 2.52 (1.76; 3.02) |  | 2.41 (2.20; 2.72) | 2.54 (2.31; 2.79) |
| 33 | 2.73 (2.46; 2.87) | 2.72 (2.45; 2.86) |  | 2.72 (2.61; 3.01) | 2.92 (2.74; 3.16) |
| 34 | 2.88 (2.30; 3.17) | 2.69 (2.15; 2.96) |  | 2.24 (1.41; 2.64) | 2.22 (1.73; 2.75) |
| 35 | 2.35 (2.12; 2.82) | 2.38 (2.14; 2.86) |  | 3.05 (2.88; 3.31) | 2.93 (2.68; 3.26) |
| 36 | 2.54 (2.29; 2.79) | 2.75 (2.48; 3.03) |  | 2.59 (1.92; 3.13) | 2.80 (2.45; 3.28) |
| 37 | 2.55 (2.30; 3.06) | 2.55 (2.30; 3.06) |  | 2.76 (2.57; 3.07) | 2.41 (2.26; 2.67) |
| 38 | 2.53 (2.28; 2.78) | 2.43 (2.19; 2.67) |  | 2.61 (2.48; 2.82) | 2.24 (2.08; 2.44) |
| 39 | 2.58 (1.81; 2.97) | 2.46 (1.72; 2.83) |  | 2.61 (2.56; 2.73) | 2.38 (2.01; 2.49) |
| 40 | 3.18 (2.71; 3.45) | 3.35 (2.96; 3.55) |  | 2.70 (2.59; 2.91) | 3.00 (2.88; 3.22) |

^1^List of cultivars is presented in Table 1.

**Supplementary Figures**


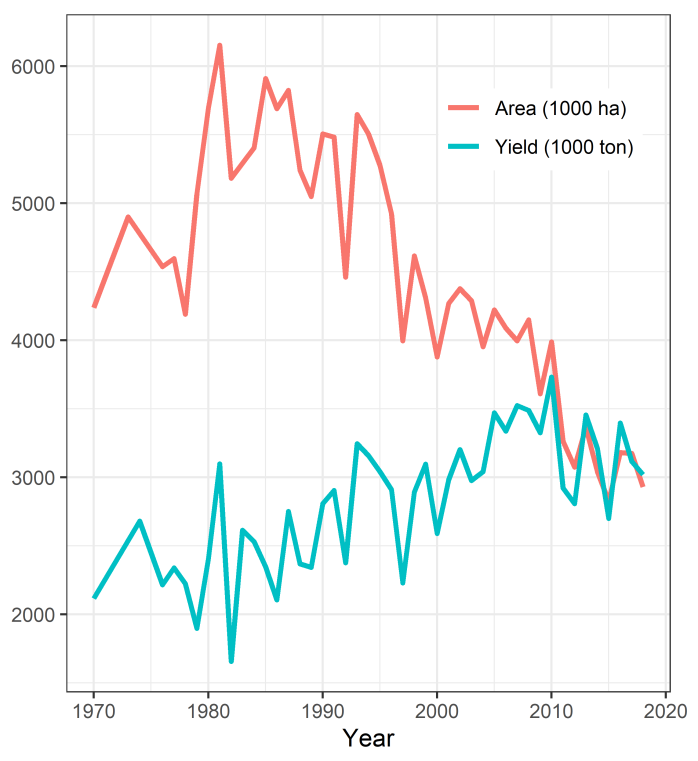


**Supplementary Figure 1.** History of common bean production and cultivated area in Brazil from 1970 to 2018. Data obtained from CONAB (2020b).

**
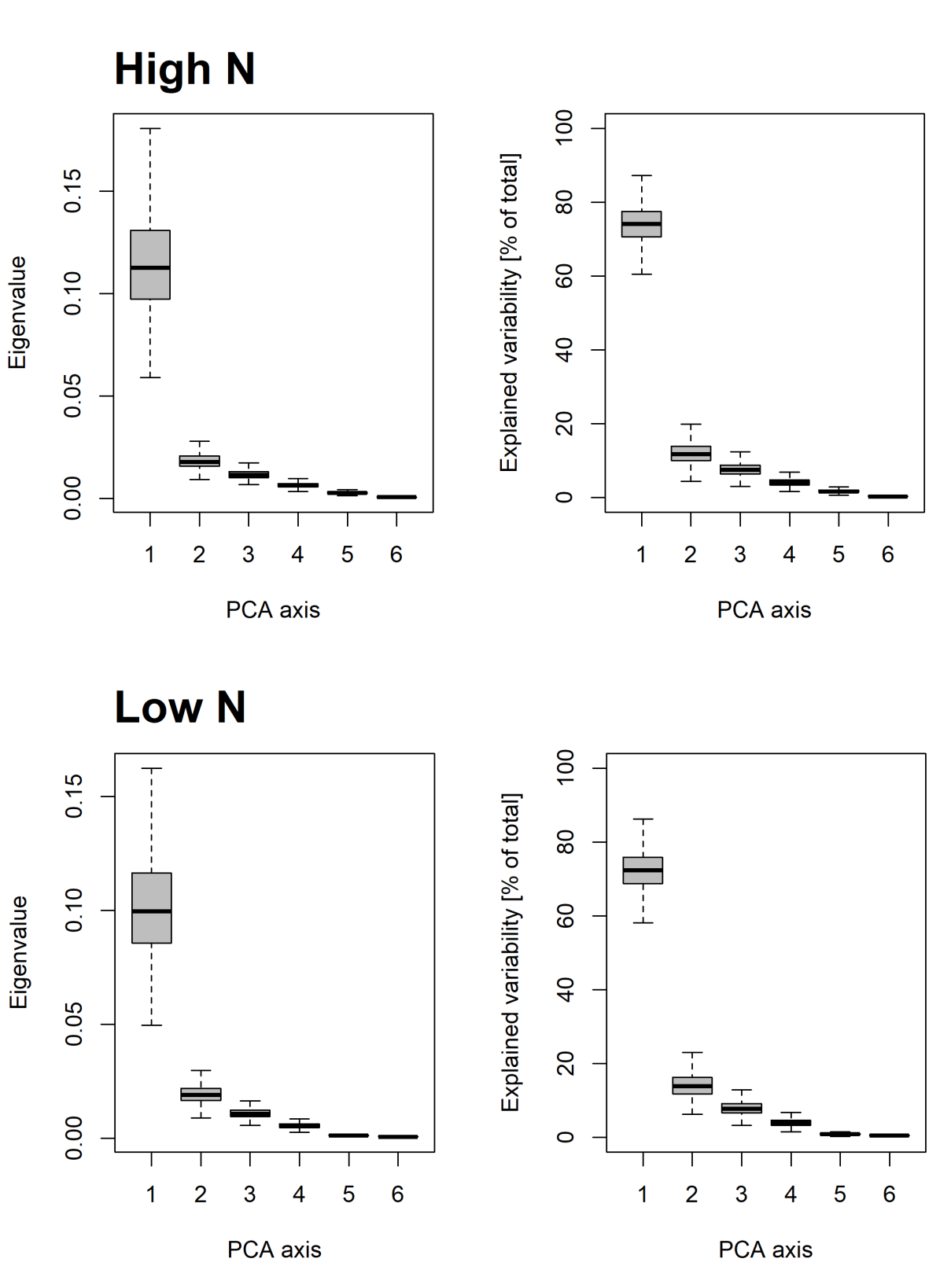
**

**Supplementary Figure 2.** Estimates of eigenvalue and total variability explained in the six axes of the principal components analysis (PCA) under high and low N conditions.

**
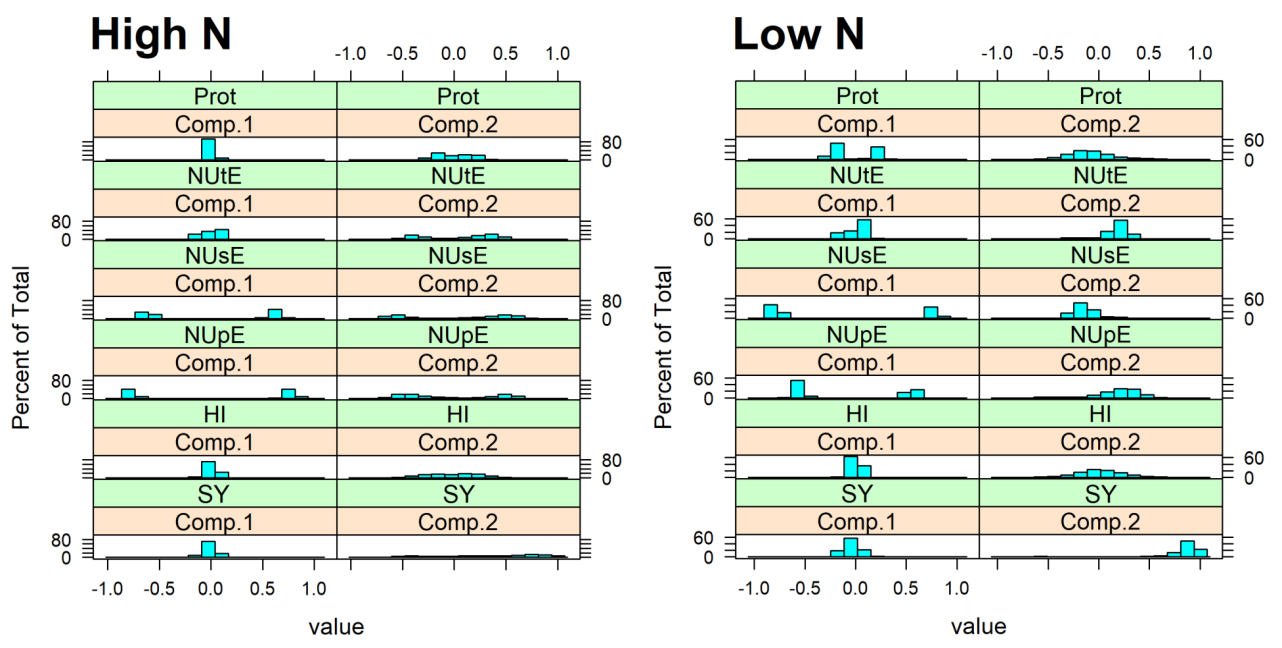
**

**Supplementary Figure 3.** Estimates of eigenvectors from the first principal components (Comp. 1 and Comp. 2, respectively) for the traits of seed yield (SY), seed protein content (Prot), harvest index (HI), nitrogen uptake efficiency (NUpE), nitrogen utilization efficiency (NUtE), and nitrogen use efficiency (NUsE) evaluated under high and low N conditions.
